# Prognostic significance of FAM3C in esophageal squamous cell carcinoma

**DOI:** 10.1186/s13000-015-0424-8

**Published:** 2015-10-24

**Authors:** Ying-Hui Zhu, Baozhu Zhang, Mengqing Li, Pinzhu Huang, Jian Sun, Jianhua Fu, Xin-Yuan Guan

**Affiliations:** Sun Yat-sen University Cancer Center, State Key Laboratory of Oncology in South China, Collaborative Innovation Center for Cancer Medicine, Guangzhou, China; Department of Clinical Oncology, Faculty of Medicine, The University of Hong Kong, Hong Kong, China; Guangdong Esophageal Cancer Institute, Guangzhou, China; Department of Thoracic Oncology, Sun Yat-sen University Cancer Center, Guangzhou, China; Department of Colorectal Surgery, The Sixth Affiliated Hospital, Sun Yat-sen University, Guangzhou, China

**Keywords:** FAM3C, ESCC, Prognosis, EMT

## Abstract

**Background:**

Family with sequence similarity 3, member C (FAM3C) has been identified as a novel regulator in epithelial-mesenchymal transition (EMT) and metastatic progression. However, the role of FAM3C in esophageal squamous cell carcinoma (ESCC) remains unexplored. The purpose of present study is to illustrate the role of FAM3C in predicting outcomes of patients with ESCC.

**Methods:**

FAM3C expression was measured in ESCC tissues and the matched adjacent nontumorous tissues by quantitative real-time RT-PCR and Western blot analysis. The relationship between *FAM3C* expression and prognosis of ESCC patients was further evaluated by univariate and multivariate regression analyses. Univariate and multivariate analyses of the prognostic factors were performed using Cox proportional hazards model.

**Results:**

The *FAM3C* mRNA expression was remarkably upregulated in ESCC compared with their nontumor counterparts (*P* < 0.001). In addition, high expression of *FAM3C* was significantly associated with pT stage (*P* = 0.014) , pN stage (*P* = 0.026) and TNM stage (*P* = 0.003). Kaplan-Meier analysis showed that the 7-year overall survival rate in the group with high expression of *FAM3C* was poorer than that in low expression group (32.0 versus 70.9 %; *P* < 0.001). Univariate and multivariate analyses demonstrated that *FAM3C* was an independent risk factor for overall survival. Moreover, Stratified analysis revealed that *FAM3C* expression could differentiate the prognosis of patients in early clinical stage (TNM stage I-II).

**Conclusions:**

*FAM3C* expression was dramatically increased in ESCC and might serve as a valuable prognostic indicator for ESCC patients after surgery.

## Background

Esophageal cancer (EC) is one of the most common malignancies and ranked as the sixth leading cause of cancer-related mortality worldwide [1, 2]. Esophageal squamous cell carcinoma (ESCC), the most prevalent histological subtype of EC, is characterized by its remarkable geographic distribution, and predominates in Northern Iran, South Africa, and Northern China, especially in Henan province [3, 4]. Despite the recent advances in diagnosis and treatment, the prognosis of ESCC patients remains dismal and the 5-year overall survival rate is ranging from 10 to 41 % [5–7]. ESCC has been viewed as a complex and heterogeneous disease which is driven by a series of genetic and epigenetic alterations. Therefore, it is imperative to search for sensitive and specific biologic markers for prevention, screening, diagnosis and development of specific therapies.

To obtain a clear picture of genetic alterations occurring in ESCC patients, our group performed high-throughput transcriptome sequencing (RNA-Seq) on three matched pairs of ESCC and the adjacent nontumorous tissues to identify differentially expressed genes. Family with sequence similarity 3, member C (FAM3C), or interleukin-like epithelial-mesenchymal transition inducer (ILEI) brought up our attention due to its significantly upregulated expression in ESCC specimens. It belongs to the family with sequence similarity 3 (FAM3) superfamily and encodes a secreted protein with a GG domain. There are four members in this family, FAM3A, FAM3B, FAM3C, and FAM3D, each encoding a protein (224–235 amino acids) with a hydrophobic leader sequence [8]. FAM3C was initially identified as a candidate gene for autosomal recessive non-syndromic hearing loss locus 17 (DFNB17) [9]. Recent works have revealed that FAM3C was a novel regulator of epithelial-mesenchymal transition (EMT) and metastatic progression [10, 11]. In addition, overexpression of FAM3C was detected in pancreatic cancer and colorectal cancer [12, 13], suggesting important roles of FAM3C in the metastasis and progression of cancer. However, the expression pattern and clinical significance of FAM3C in ESCC has not been explored.

Here we measured the expression level of FAM3C in ESCC and matched adjacent nontumorous specimens, and further explored its clinicopathological significance and prognostic value in ESCC.

## Methods

### Patients and tissue samples

One hundred seven primary ESCC tumor and 40 paired adjacent nontumorous tissue samples were collected immediately after surgery resection at Sun Yat-sen University Cancer Center between March 2002 and October 2008. The inclusion criteria were as follows: (a) definitive ESCC diagnosis by pathology based on WHO criteria; (b) complete surgical resection, defined as complete resection of all tumor nodules with the cut surface being free of cancer by histologic examination; (c) no neoadjuvant or adjuvant treatment before surgery; (d) complete clinicopathologic and follow-up data. Ethical approval for this study was granted by the Medical Ethics Committee of Sun Yat-sen University Cancer Center. All patients signed informed consent. Tumor differentiation (G1, well differentiated; G2, moderately differentiated; G3, poorly differentiated), depth of tumor invasion (pT stage) and lymph node metastasis (pN stage) were determined by pathologic examination. Tumor staging was determined according to the seventh edition tumor-node-metastasis (TNM) classification of the American Joint Committee on Cancer [14].

### Quantitative real-time reverse transcription polymerase chain reaction (qRT-PCR)

All fresh tumorous and nontumorous tissue samples were taken from regions which macroscopically judged to be neoplastic and normal, respectively. Both of them were immediately stored at dry ice after resection and then frozen at −80 °C. Total RNA was extracted from clinical samples using TRIzol reagent (Invitrogen), and was reverse-transcribed using an Advantage RT-for-PCR Kit (Clontech Laboratories) according to the manufacturer’s instructions. qRT-PCR was performed to detect mRNA levels of the corresponding glyceraldehyde-3-phosphate dehydrogenase (*GAPDH*), *FAM3C*, *E-cadherin* and *vimentin* using a SYBR Green PCR Kit (Applied Biosystems) and LightCycler480 384-well PCR system (Roche Diagnostics). The *GAPDH* was used as an internal control for *FAM3C*, *E-cadherin* and *vimentin*. Primers for *FAM3C* are 5′-CCTTGGCAAATGGAAAAACAGG-3′ (forward) and 5′-CCCAAATCAGCAATGAGCCG-3′ (reverse). Primers for *E-cadherin* are 5′-TGAAGGTGACAGAGCCTCTGGAT-3′ (forward) and 5′-TGGGTGAATTCGGGCTTGTT-3′ (reverse). Primers for *vimentin* are 5′-CCTTGAACGCAAAGTGGAATC-3′ (forward) and 5′-GACATGCTGTTCCTGAATCTGAG-3′ (reverse). Primers for *GAPDH* are 5′-ACTTCAACAGCGACACCCACTC-3′ (forward) and 5′-TACCAGGAAATGAGCTTGACAAAG-3′ (reverse). The value of relative expression for each sample was averaged and compared using the Ct method. *ΔΔ*Ct(sample) = *Δ*Ct(sample) - *Δ*Ct(calibrator), *Δ*Ct(sample) = Ct(sample) of target gene - Ct(sample) of *GAPDH*; *Δ*Ct(calibrator) = Ct(calibrator) of target gene - Ct(calibrator) of *GAPDH*; calibrator was defined as the pooled samples from 40 adjacent nontumorous tissues. The fold changes in mRNAs were calculated by the equation 2^–*ΔΔ*Ct^.

### Western blot analysis

Frozen tissue specimens were ground under liquid nitrogen. Total protein was extracted with lysis buffer for one hour on ice. Equal amounts of protein were separated by 15 % SDS-PAGE and electrophoretically transferred to polyvinylidene difluoride membranes (Roche) using a mini trans-blot apparatus (Bio-Rad Laboratories). Membranes were blocked with TBS-0.1 % Tween 20 containing 5 % nonfat dry milk for one hour at room temperature and incubated with rabbit polyclonal antibody against FAM3C (1:1,000; Proteintech) or GAPDH (1:1,000; Abgent) at 4 °C overnight. Membranes were then washed three times with TBS-0.1 % Tween 20 and incubated with horseradish peroxidase (HRP) –conjugated goat anti-rabbit IgG antibody (Cell Signaling Technology) at a 1:3,000 dilution for one hour at room temperature. Blots were developed using a Luminata Crescendo Western HRP substrate (Millipore). GAPDH was used as a loading control.

### Statistical analysis

All statistical analyses were performed using the Statistical Package for the Social Sciences (SPSS) version 16.0 (SPSS Inc, Chicago, IL). Paired two-tailed student’s *t* test was used to compare the expression of *FAM3C* in primary ESCC tumors and their corresponding adjacent nontumorous tissues. The correlation between *FAM3C* expression and clinicopathological parameters was assessed by chi-square test or Fisher’s exact test. Overall survival (OS) was defined as the interval from curative surgery to either the time of death from ESCC or last follow up (30 June 2015). The prognostic value was calculated by the Kaplan-Meier analysis with log-rank test. Univariate and multivariate survival analyses were performed using the Cox proportional hazard model with a forward stepwise procedure (the entry and removal probabilities were 0.05 and 0.10, respectively). A significant difference was considered statistically when *P* value was < 0.05.

## Results

### Expression of *FAM3C* in ESCC

**Fig. 1:**
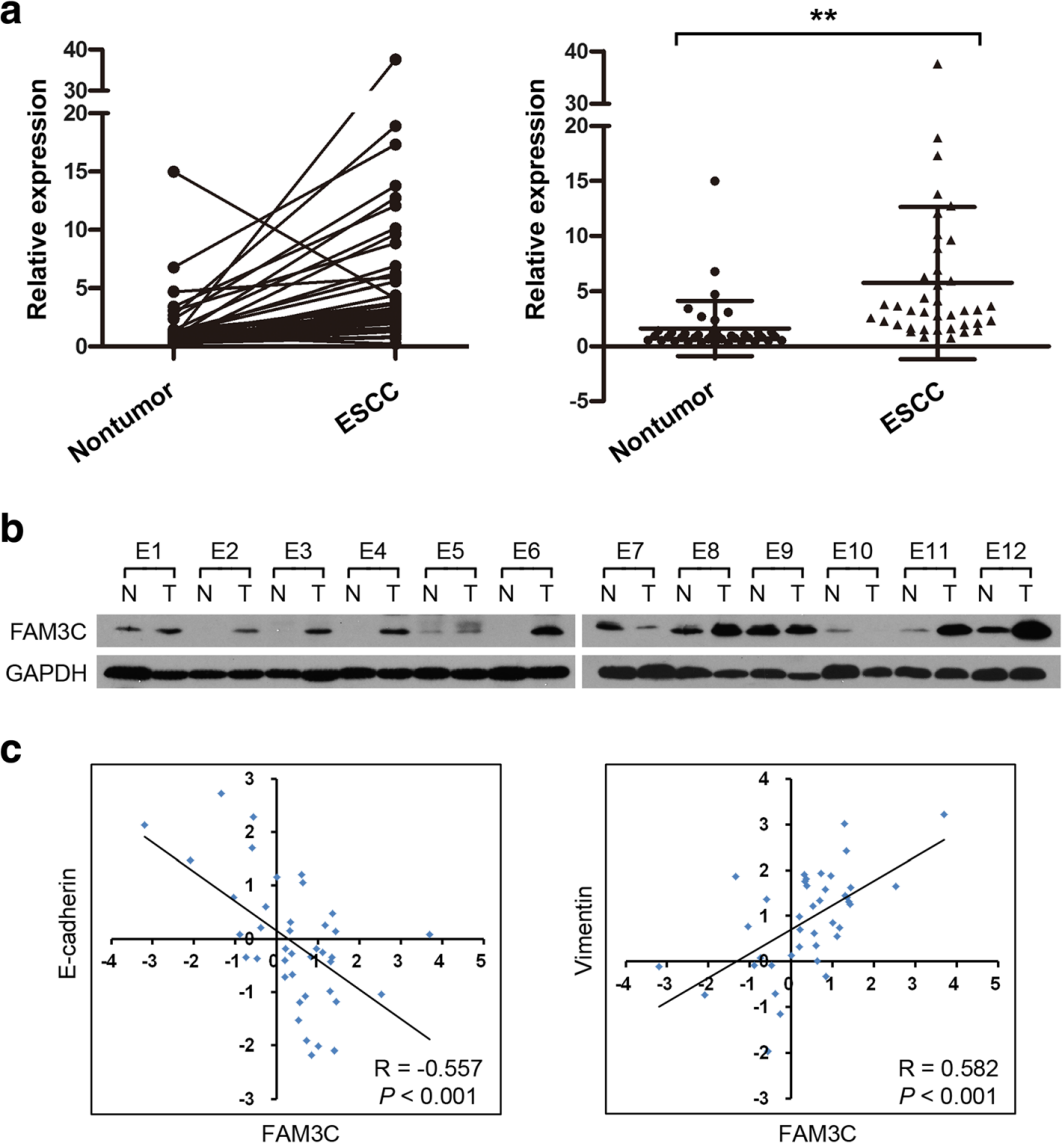
Upregulation of *FAM3C* in ESCC. **a** *FAM3C* mRNA expression in ESCC and matched adjacent nontumor specimens from 40 cases of ESCC patients. The dot plots represent the *FAM3C* expression in tumor and nontumor samples from 40 ESCCs. **, *P* < 0.001, paired *t*-test. **b** Western blot analysis of FAM3C expression levels in 12 paired ESCC and nontumor specimens. GAPDH was used as a loading control. N, nontumor tissue; T, tumor tissue. **c** Analysis showing linear regressions and significant Pearson correlations of *FAM3C* with *E-cadherin* and *FAM3C* with vimentin in 40 ESCC samples

Our previous RNA-seq data showed that *FAM3C* was overexpressed in all three tested ESCC tumor tissues compared to corresponding nontumor tissues. The mRNA expression of *FAM3C* was initially tested in 40 pairs of primary ESCC tumors and their normal counterparts by qRT-PCR. Elevated expression of *FAM3C* (defined as > 2-fold change) was detected in 28 of 40 (70.0 %) of ESCC tissues compared with the matched nontumorous tissues (Fig. 1a). The average level of *FAM3C* expression in tumor specimens was dramatically higher than that in nontumor specimens (5.72 versus 1.61, *P* < 0.001, paired Student’s *t* test; Fig. 1a). To confirm our findings, Western blot analysis of FAM3C expression was performed in the paired ESCC and the nontumor specimens of 12 randomly selected ESCCs from the 40 cases described above. Consistently, the upregulation of FAM3C protein was observed in 8 of 12 ESCC tissues compared with their matched nontumor specimens (Fig. 1b).

### Correlation of *FAM3C* mRNA expression with EMT markers

To determine the correlation between the expression of FAM3C and EMT, expression patterns of FAM3C and EMT-associated markers, including E-cadherin and vimentin, were assessed by qRT-PCR in 40 ESCC specimens described above. Linear regression analyses showed that the expression of *FAM3C* was negatively correlated with *E-cadherin* expression (R = −0.557, *P* < 0.001), but positively correlated with *vimentin* expression (R = 0.582, *P* < 0.001; Fig. 1c).

### Correlation of *FAM3C* mRNA expression with clinicopathological variables

Subsequently, we examined the correlation between the expression of *FAM3C* and the clinicopathological features of ESCC. High level expression of *FAM3C* was detected in 71/107 (66.4 %) of informative ESCC tissues. The median fold change of *FAM3C* (2.28) in ESCC tumor specimens was used as a cutoff value to divide all 107 patients into two groups: high expression group (*n* = 53) and low expression group (*n* = 54). As showed in Table 1, high expression of *FAM3C* was significantly associated with pT stage (*P* = 0.014), pN stage (*P* = 0.026) and TNM stage (*P* = 0.003). No correlation was observed between *FAM3C* expression and other clinicopathological index.

**Table 1:** Association of *FAM3C* expression with clinicopathological features in ESCC

Clinical features	Cases	*FAM3C* mRNA expression		*P* value
		low level (%)	high level (%)	
Age (years old)				0.204
≤59	49	28 (57.1 %)	21 (42.9 %)	
>59	58	26 (44.8 %)	32 (55.2 %)	
Gender				0.954
Male	79	40 (50.6 %)	39 (49.4 %)	
Female	28	14 (50.0 %)	14 (50.0 %)	
Location				0.811
Upper	12	5 (41.7 %)	7 (58.3 %)	
Middle	60	31 (51.7 %)	29 (48.3 %)	
Lower	35	18 (51.4 %)	17 (48.6 %)	
Differentiation				0.843
Grade 1	20	9 (45.0 %)	11 (55.0 %)	
Grade 2	57	29 (50.9 %)	28 (49.1 %)	
Grade 3	30	16 (53.3 %)	14 (46.7 %)	
pT stage				**0.014**
T1-2	25	18 (72.0 %)	7 (28.0 %)	
T3-4	82	36 (43.9 %)	46 (56.1 %)	
pN stage				**0.026**
N0	56	34 (60.7 %)	22 (39.3 %)	
N1-3	51	20 (39.2 %)	31 (60.8 %)	
TNM stage				**0.003**
I-II	60	38 (63.3 %)	22 (36.7 %)	
III	47	16 (34.0 %)	31 (66.0 %)	

Statistical significance (*P* < 0.05) is shown in bold

### *FAM3C* mRNA expression and patient outcomes

**Fig. 2:**
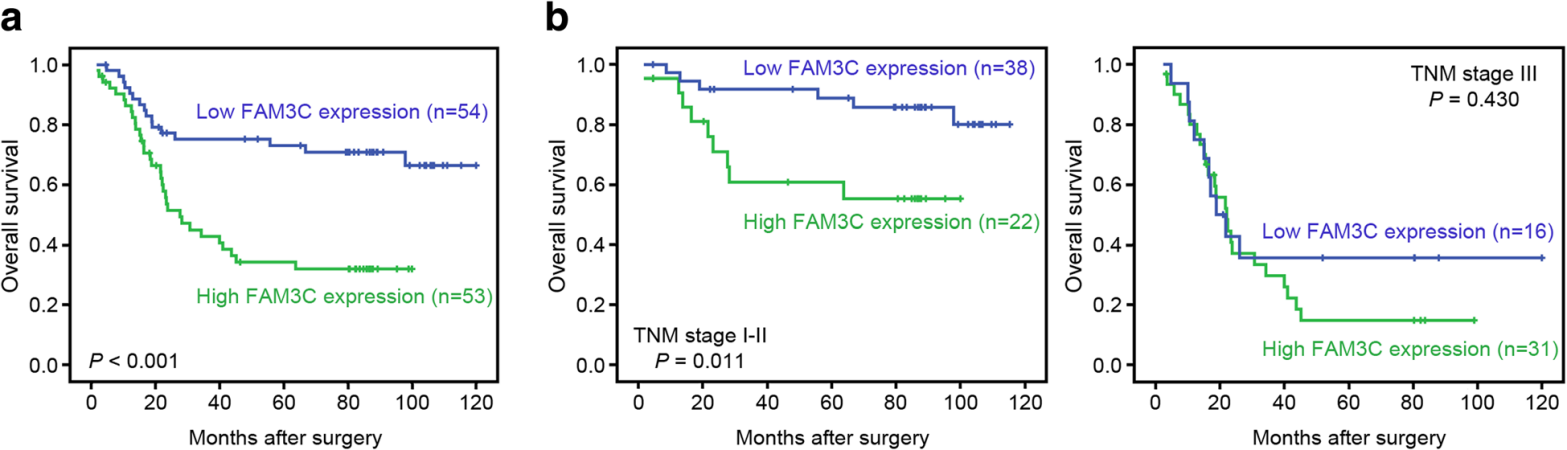
Kaplan-Meier analysis of OS for *FAM3C* expression. **a** Kaplan-Meier analysis of OS for *FAM3C* expression in 107 cases of ESCC patients. **b** Stratified survival analysis according to the TNM stage

The association between *FAM3C* expression and prognosis of ESCC patients was investigated by Kaplan-Meier analysis and log-rank test. As shown in Fig. 2a, ESCC patients with high *FAM3C* expression had poorer OS than those with low *FAM3C* expression. The 1, 3, and 7-year OS rate in the high *FAM3C* expression group were 82.5, 42.9, and 32.0 %, respectively, compared with 88.7, 75.3, and 70.9 %, respectively, in the low *FAM3C* expression group (log-rank test, *P* < 0.001). In the univariate analysis, the statistically significant predictors for a patient’s OS were cell differentiation (*P* = 0.025), pT stage (*P* = 0.003), pN stage (*P* < 0.001), and *FAM3C* expression (*P* < 0.001) (Table 2). In the multivariate Cox analyses, cell differentiation (*P* = 0.043), pT stage (*P* = 0.045), pN stage (*P* < 0.001), and *FAM3C* expression (*P* = 0.022) were shown to be the independent prognostic predictors for OS (Table 2). Further, in a stratified survival analysis according to the TNM stage, *FAM3C* expression could differentiate the prognosis of patients with TNM stage I-II (*P* = 0.011; Fig. 2b).

**Table 2:** Cox proportional hazard regression analyses for overall survival

Clinical features	Univariate analysis		Multivariate analysis	
	HR (95 % CI)	*P* value	HR (95 % CI)	*P* value
Age	1.321 (0.747–2.337)	0.338	–	–
Gender	0.613 (0.297–1.265)	0.186	–	–
Location	1.012 (0.647–1.583)	0.958	–	**–**
Differentiation	1.574 (1.014–2.443)	**0.025**	1.659 (1.016–2.708)	**0.043**
pT stage	5.922 (1.838–19.089)	**0.003**	3.463 (1.029–11.661)	**0.045**
pN stage	5.470 (2.864–10.447)	<**0.001**	3.789 (1.937–7.409)	<**0.001**
*FAM3C* expression	2.941 (1.606–5.389)	<**0.001**	2.067 (1.109–3.854)	**0.022**

*HR* Hazard ratio, *CI* Confidence interval

Statistical significance (*P* < 0.05) is shown in bold

## Discussion

EMT plays pivotal roles during tumor progression through endowing cells with migratory and invasive properties, inducing stem cell properties, and preventing apoptosis and senescence [15]. FAM3C was regarded as a key regulator of EMT and metastatic progression in both human and mouse models [10, 11]. In the current study, we found that 70.0 % of ESCC patients showed elevated *FAM3C* expression in their tumor tissues compared with the normal counterparts. Our results also showed that the expression of *FAM3C* was associated with the expression of *E-cadherin* and *vimentin*, which are the vital factors in the process of EMT. Further, the genetic-clinicopathologic correlation analysis indicated that patients with high expression of *FAM3C* in tumorous specimens tended to have more advanced pT stage, pN stage and a higher TNM stage. These findings suggest that FAM3C may initiate EMT process, and thus contributing to ESCC metastasis and progression. Consistent with our results, recent investigations demonstrated that overexpression of FAM3C correlated with EMT and metastasis in breast cancer and colon cancer [10, 13].

EMT, a switch of polarized epithelial cells to a highly motile mesenchymal phenotype, is a developmental event recognized as a central process during cancer progression and metastasis [16, 17]. TGF-β has been implicated as a “master switch” in EMT process, which regulates expression of numerous downstream transcription factors involved in EMT without or with the collaboration of other signaling effectors [18, 19]. Several works revealed that TGF-β-induced EMT was mediated through the induction of FAM3C in murine epithelial cells [11, 20, 21]. Heterogeneous nuclear ribonucleoprotein (hnRNP E1) repressed FAM3C translation by binding to a TGF-β-activated translation (BAT) element in the 3′UTR of FAM3C. The activation of TGF-β induced phosphorylation at Ser43 of hnRNP E1 by protein kinaseBβ/Akt2, which resulted in its release from the BAT element and thus reversed the translation inhibition of FAM3C [11, 20, 21]. Recently, a similar translational regulation pattern of FAM3C was observed in human lung cancer cell line A549 [22]. These data indicated that FAM3C was regulated by post-translational modification during EMT. Besides, our initial RNA-Seq profiling data and subsequent qRT-PCR analysis demonstrated that the expression of *FAM3C* was upregulated at RNA level in ESCCs. *FAM3C* was located on chromosome 7q31. Amplification of 7q is one of the most frequent allelic imbalances in ESCC detected by comparative genomic hybridization (CGH) [23, 24], suggesting the existence of one or more ESCC-related oncogenes within this region. Accordingly, it raises the possibility that the gains in 7q may contribute to the overexpression of *FAM3C* mRNA in ESCC. Based on these findings, we speculate that the expression of FAM3C may be regulated via multiple mechanisms including post-transltional modification, DNA copy number change, hypermethylation, histone deacetylation, miRNA regulation, etc. Further elucidation for the precise mechanism underlying the regulation of FAM3C expression in ESCC is required.

Early research demonstrated that FAM3C alone was sufficient to induce EMT, tumor growth and metastasis in murine mammary epithelium cell EpH4, independently of TGF-β activation [10]. However, a recent research revealed that exogenous FAM3C strictly required co-operation with oncogenic Ras to cause TGF-β-independent EMT and tumor progression in human hepatocytes [25]. These results suggest that the underlying mechanisms of FAM3C involved in EMT may vary depending on the epithelial cell type and tissue context. Moreover, the result that endogenous, secreted FAM3C-induced EMT could be partially suppressed by a neutralizing antibody against FAM3C in EpRas cells [10], implies that FAM3C may induce multiple autocrine growth factors and chemokines loops to cause TGF-β-independent EMT. Interestingly, activation of autocrine PDGF/PDGF-R signaling was observed in both FAM3C-induced murine mammary epithelial EMT and RAS/FAM3C-induced hepatocellular EMT [10, 25]. This raises the question of whether FAM3C acts on EMT-associated autocrine loops or the specific FAM3C receptors. Nevertheless, until now, the precise FAM3C-dependent signal transduction pathways or receptors involved in EMT are not completely understood.

More importantly, our data demonstrated that elevated expression of *FAM3C* was significantly associated with poor OS of ESCC patients. Patients with high *FAM3C* expression displayed a remarkably lower rate of 7-year OS than those with low *FAM3C* expression. Moreover, with the stratified survival analysis according to the TNM stage, we found that high expression *FAM3C* could identify the subgroup of patients with poor outcomes among the early clinical stage (TNM stage I-II) cases, but not the advanced clinical stage ( TNM stage III). Metastasis is regarded as a multistep process characterized by dissociation of tumor cells from adjacent normal cells, penetration into the underlying interstitial matrix, intravasation, survival in the circulation, extravasation at a distant organ site and growth of metastatic cells in the distant organ [26]. Accumulating evidence indicates that EMT is involved in the early steps of metastasis [27, 28]. Overexpression of FAM3C may induce the incipient ESCC cells to undergo EMT and subsequently acquire invasive and migratory abilities, which leads to the poor prognosis in early clinical stage. The overall survival rate of patients with advanced clinical stage remains dismal, which is attributed to metastatic relapse after resection of the primary tumor. According to the metastasis model, only the cancer cells that adopt various strategies can be survive and eventually outgrow in the target organ. In addition, systemic signals, which act directly or indirectly on the microenvironment in which metastases arise, have impacts on latter steps in the metastatic cascade [29, 30]. Hence, the prognostic significance of FAM3C did not retain in advanced clinical stage. Together, data from the current study imply that combining *FAM3C* mRNA expression and clinicopathological variables may predict outcomes of patients with early pathological stage more accurately. Accordingly, postoperative adjuvant therapy or careful follow-up may be recommended for this subgroup of patients to improve the postoperative outcomes. However, further studies are required with larger sample sizes to validate these findings.

## Conclusions

In the present study, we, for the first time, reported that *FAM3C* expression was upregulated in ESCC, which was associated with aggressive tumor behavior, metastasis, and poor clinical outcome. Therefore, *FAM3C* may be a valuable biomarker for the prediction of ESCC prognosis.

## Abbreviations

FAM3C: Family with sequence similarity 3, member C
ILEI: Interleukin-like epithelial-mesenchymal transition inducer
FAM3: Family with sequence similarity 3
EC: Esophageal cancer
ESCC: Esophageal squamous cell carcinoma
OS: Overall survival
TNM: Tumor-node-metastasis
RNA-Seq: Transcriptome sequencing
qRT-PCR: Quantitative real-time reverse transcription polymerase chain reaction
GAPDH: Glyceraldehyde-3-phosphate dehydrogenase
EMT: Epithelial-mesenchymal transition
TGF-β: Transforming growth factor beta
hnRNP E1: Heterogeneous nuclear ribonucleoprotein E1
BAT: TGF-β-activated translation
CGH: comparative genomic hybridization
